# Towards an increased attention on ADHD symptoms and traits in young adults: prevalence data from screening tools in a psychiatric outpatient clinic

**DOI:** 10.1192/j.eurpsy.2024.1180

**Published:** 2024-08-27

**Authors:** C. Sanguineti, V. Nistico’, I. Folatti, G. Santangelo, R. Faggioli, A. Bertani, O. Gambini, B. Demartini

**Affiliations:** ^1^Health Science Department, University of Milan; ^2^Department of Psychology, University of Milan Bicocca; ^3^ Aldo Ravelli Research Center for Neurotechnology and Experimental Brain Therapeutics, University of Milan; ^4^UO Psichiatria 51 e 52; ^5^Centro Giovani Ettore Ponti, ASST Santi Paolo e Carlo, Presidio San Paolo, Milan, Italy

## Abstract

**Introduction:**

Attention–Deficit/Hyperactivity Disorder (ADHD) is a heterogeneous neurodevelopmental disorder encompassing developmentally inappropriate inattentiveness, hyperactivity, and increased impulsivity (DSM-5). Symptoms presentation is different for different stages of life; moreover, individuals with ADHD symptomatology can develop abilities and strategies that help them adapt and mask the distinctive features of the condition, thus reducing the functional impairment usually seen in ADHD subjects, and ultimately not receiving neither a clinical diagnosis nor a proper therapeutic support. They might express their lack of well-being through other transdiagnostic symptoms, and finally reach psychiatric attention for potential comorbidities. Hence, it was argued that the existence of children and adolescents with subthreshold and underrecognized symptoms that subsequently develop into a full diagnosis suggests that ADHD should be significantly more considered in adult mental health settings.

**Objectives:**

Here we aimed to analyse the prevalence of ADHD symptoms and traits in a heterogeneous clinical psychiatric sample of young adults (aged 18 to 24 years old), who referred to a specialized outpatient clinic for various psychiatric and psychological disturbances.

**Methods:**

259 participants completed three validated self-report screening questionnaires for ADHD: the Wender Utah Rating Scale (WURS), the Adult Attention-Deficit/Hyperactivity Disorder Self-Report Screening Scale for DSM-5 (ASRS-5), and the Conners’ adult ADHD rating scale (CAARS).

**Results:**

12.4% of our sample scored above the cut-off at both the WURS and the ASRS-5 and was considered at risk of ADHD.

**Conclusions:**

The prevalence rate in our sample is higher than the one found in the adult general population (6.76%), and in the lower range of the one found in the adult clinical population (6.9% to 38.8%). We discuss the potential role of sociodemographics (age, sex, gender identity, and employment) and comorbidity factors. Differences in the clinical presentation of ADHD according to sex assigned at birth and age should be considered during every psychiatric evaluation to minimize the risk of underdiagnosis. We advocate for further studies investigating the prevalence of ADHD in different psychiatric services for adults, and for a stronger presence of specialistic ADHD services and trained clinicians on the territory: this would increase diagnostic reliability, consequently providing a better treatment for ADHD in adults, and facilitate the transition from pediatric to adult’s services.

**Disclosure of Interest:**

None Declared

